# Low-Intensity Pulsed Ultrasound Therapy in Patients With Post-traumatic Delayed Union and Non-union

**DOI:** 10.7759/cureus.32267

**Published:** 2022-12-06

**Authors:** Jacques Pretorius, Marzanne Barry, Ashraf Fadul, Colin G Murphy

**Affiliations:** 1 Trauma and Orthopaedics, University Hospital Galway, Galway, IRL

**Keywords:** non-surgical management, exogen™, delayed osseous union, non-union, low intensity pulsed ultrasound (lipus)

## Abstract

Background

Fracture non-union can lead to significant patient morbidity with poor quality of life. Due to the cost, complexity, and potential risks of revision surgery, there has been an increased popularity in the use of low-intensity pulsed ultrasound therapy (LIPUS), which accelerates and promotes bone consolidation. There is an ongoing debate regarding the use and efficacy of LIPUS in delayed union and non-union. This study aims to assess the success rate of LIPUS therapy in patients treated for delayed and non-union fractures, explicitly focusing on the impact of patient co-morbidities and fracture characteristics.

Method

A retrospective observational study was performed of all consecutive patients who received LIPUS therapy in a single institution from January 2016 to September 2022. Of 127 identified patients, only 99 patients met our inclusion criteria. Data collection entailed reviewing the clinical notes to assess patients' sex, age, co-morbidities, initial treatment method, time to initiate LIPUS, whether a CT was performed to diagnose non-union, time to union and whether revision surgery was needed. Two independent senior orthopedic doctors reviewed the patients' radiographs, measured the interfragmentary bone gap of all fractures, and assessed whether the radiographic union was achieved.

Results

The mean age of the included patients was 52.5 (SD±16.9) years with a male-to-female ratio of 1:1.6. At initial presentation, 65 (out of 99) patients were treated surgically, whereas the rest were managed conservatively. 80.8% of patients developed atrophic non-union. All 99 included patients were fitted with LIPUS once delayed/ non-union was diagnosed; the average time to fitting was 5.1 (SD±3.9) months. Of these, 61.6% of patients were successfully treated with LIPUS with a clinical and radiological union at an average of 4.3 (SD±1.9) months. The rest of the patients needed further surgical intervention due to ongoing non-union. The interfragmentary bone gap was the only statistically significant factor influencing the success of LIPUS therapy (p=0.003). In contrast, no statistically significant association was identified between the outcome of LIPUS therapy and the patient's age, sex, diabetes, and smoking status.

Conclusion

This study demonstrated a 61.6% progression to union rate of patients treated with LIPUS therapy for delayed union and non-union. The interfragmentary bone gap was identified as the only statistically significant factor influencing the success of LIPUS therapy. In the current climate post-lockdown and with ongoing Covid 19 outbreaks impacting elective waiting lists negatively, there is increased value and demand for non-surgical treatment options. LIPUS therapy represents an important complementary non-surgical and low-risk treatment pathway for delayed union and non-union.

## Introduction

Although bone has an excellent healing and remodeling potential [1], it has been identified that there is a 5 to 10% risk of developing fracture non-union [2,3]. The risk of complicated bone healing increases dramatically with age [4]. The increase in an aging population underscores the importance of advances in novel technologies to support fracture regeneration [5] and highlights the importance of technically accomplished surgery in the first iteration. Another factor that might be contributed to the increased number of non-union is the improved survival rate of patients with severe injuries [6]. The risk of developing non-union is multifaceted, including fracture severity, location, co-morbidities, medication, and surgical technique [7].

The current gold standard for non-union is revision surgery, which entails decortication of the edges, autologous bone graft, and definitive fixation. Revision surgery is not without risks and does not guarantee success - with an average union rate of 68% to 96% [8]. Non-unions also have a significant socio-economic impact on patients and the healthcare system [9]. Avoiding revision surgery can substantially benefit both the patient and the healthcare system.

A well-known non-invasive treatment option for delayed union and non-union includes low-intensity pulsed ultrasound (LIPUS) therapy. Low-intensity pulsed ultrasound therapy (LIPUS) has been used to treat fracture non-union in the clinical setting for over 20 years [6]. This was first demonstrated to be a potential treatment option to assist fracture regeneration by Duarte in 1983 when he demonstrated increased callus formation in an osteotomised rabbit fibula and femur-stimulated by LIPUS [10]. Rutten et al. furthermore demonstrated that LIPUS stimulates bone regeneration by increasing osteoblast activity by performing a quantitative histometric analysis of biopsy samples from delayed fibular unions. This study showed a significant increase in osteoid thickness, mineral apposition rate, and bone volume [11]. The use of LIPUS was reviewed by the national institute of clinical excellence (NICE), which estimated that the cost saving would be £2407 per patient when treated with LIPUS for non-union instead of revision surgery [12].

This study aimed to assess the success rate of LIPUS therapy in patients treated for delayed union and non-union, explicitly focusing on the impact of patient co-morbidities and fracture characteristics.

## Materials and methods

A retrospective observational study was performed of all consecutive patients who received LIPUS therapy in a single institution from January 2016 to September 2022. Ethical approval was obtained from the clinical research ethics committee at Galway University Hospital. All cases were reviewed to assess whether they adhered to the inclusion criteria, which included all adult patients who received LIPUS therapy for fractures with delayed union or non-union. Delayed union is generally said to occur after three months with no radiological evidence of healing, and non-union is established after nine months [12]. Exogen (Bioventus, Durham, North Carolina) is the brand of LIPUS available in our region and was used for all cases in this study. Patients were excluded when they had any of the following criteria. (1) LIPUS initiated in acute fractures: less than three months. (2) Infected cases. (3) Insufficient notes. (4) Loss to follow up. (5) Previous LIPUS therapy provided. (6) LIPUS provided for fusion surgery. (7) Patient receiving LIPUS therapy for more than one site.

Data collection entailed reviewing the clinical notes to assess patients' sex, age, co-morbidities, initial treatment method, time to initiate LIPUS, whether a CT was performed to diagnose non-union, time to union and whether revision surgery was needed. Two independent senior orthopedic doctors reviewed the patients' radiographs, measured the interfragmentary bone gap of all fractures, and assessed whether the radiographic union was achieved. Radiographic union was defined as bridging callus at 3 out of 4 cortices on two orthogonal views. This was correlated with a clinical union collected from the patient notes and entailed that the patient reported no further functional pain, with a return to normal activities of daily living.

Statistical analysis was carried out using SPSS software version 21.0. Fisher exact or chi-square tests were used to show the inter-relationship between the two groups as our dataset is categorical: successful LIPUS therapy and failed LIPUS therapy (need for revision surgery). The independent sample t-test was used to evaluate the significance of the interfragmentary bone gap, and a p-value of <0.05 was considered statistically significant.

## Results

Out of 127 patients identified as having undergone LIPUS therapy, 99 met the inclusion criteria and underwent data analysis. The mean age of the included patients was 52.5 (SD±16.9) years with a male-to-female ratio of 1:1.6. At initial presentation, 65 of the 99 patients were treated surgically. In contrast, the rest were managed with conservative measures. All included patients were determined to have developed delayed or non-union and were initiated on LIPUS therapy. Atrophic non-union was the dominant type identified (80.8%). No association was found between the success of LIPUS therapy and the type of non-union (p=0.449) (Table 1). A CT was performed in 37.3% of cases to confirm and classify the type of non-union.

**Table 1:** Key factors investigated for potential association with success/ failure of LIPUS therapy n = number of patients; LIPUS = low-intensity pulsed ultrasound; mm = millimetre

Demographics		Union after LIPUS N(%)	Revision needed N(%)	P-value
Gender	Male	27 (44)	17 (45)	0.432
	Female	34 (56)	21 (55)	
Diabetes	Yes	8 (13)	6 (16)	0.835
	No	53 (87)	32 (84)	
Smoker	Yes	9 (15)	3 (8)	0.565
	No	19 (31)	14 (37)	
	Unknown	33 (54)	21 (55)	
Non-Union	Atrophic	47 (77)	33 (87)	0.449
	Hypertrophic	14 (23)	5 (13)	
Initial management	Surgery	43 (71)	22 (58)	0.276
	Conservative	18 (29)	16 (42)	
Interfragmentary Gap	Length in mm	3.2	7.6	0.003

The success rate demonstrated in this study of patients treated for delayed and non-union with LIPUS therapy was 61.6%. The patients who did not manage to heal on LIPUS therapy underwent further surgical management. The average time to fitting LIPUS for all included patients was 5.1 months (SD±3.8). The group successfully managed with LIPUS therapy was initiated on LIPUS at an earlier stage than the failed group, with an average time to the fitting of 4.4 (SD±2.3) months and 6.6 (SD±5.2) months, respectively. The patients initially treated surgically had a higher success rate on LIPUS therapy of 66.2%, whereas only 53.0% of the patients treated conservatively responded to LIPUS therapy. However, this disparity was statistically non-significant, with a p-value of > 0.05 (Table 1).

The most common sites involved in fracture non-union were tibia/fibula fractures and humerus fractures, with a prevalence of 33.3% and 24.2%, respectively (Table 2). The average interfragmentary gap was 3.2mm (SD± 2.2) for the healed fractures, whereas the interfragmentary bone gap for the failed treatment group was 7.6mm (SD± 6.8). This was determined to be a statistically significant factor (p=0.003) influencing the success of LIPUS therapy and fracture union. No statistically significant association was identified between the success of LIPUS therapy and the patient's age, sex, diabetes, and smoking status (Table 1).

**Table 2:** Distribution of fracture sites with success rates Tib/fib = Tibia/ fibula; N = number of patients

Site	Total	Union (%)	Non-Union (%)
Tib/Fib	33	17 (52)	16 (48)
Humerus	24	18 (75)	6 (25)
Foot	15	11 (73)	4 (27)
Femur	8	5 (63)	3 (37)
Clavicle	8	5 (63)	3 (37)
Hand	6	3 (50)	3 (50)
Forearm	5	2 (40)	3 (60)
All sites	99	61 (61)	38 (39)

## Discussion

LIPUS therapy has been investigated for use in fresh fractures, delayed union, and non-union with conflicting results. A systematic review and meta-analysis by Bashardoust et al. reported that LIPUS could stimulate radiologically evident bone healing in fresh fractures. In contrast, the evidence is limited, supporting its use in delayed unions and non-unions [13]. Recently a systematic review was performed, including 13 studies looking at the efficiency of LIPUS therapy in non-union, which demonstrated a success rate of > 80% and subsequently concluded that LIPUS can be considered an alternative treatment option for established non-unions [5]. Furthermore, Leighton et al. performed another systematic review identifying all the evidence available regarding the use of LIPUS in patients with infected, instrumented, and fragility fracture non-unions, which concluded that all three sub-groups produced healing rates comparable to the results observed in general non-union literature [14]. In contrast, recently, a few published retrospective studies produced low success rates of 32.8% - 57% [15-17]. In one of the few randomized controlled trials published on LIPUS therapy as an alternative to surgical management, there was no significant difference between the success rates of the placebo and intervention groups [18]. The success rate achieved in this study is 61.6%. Although this could be considered a low success rate, it is still important to note that more than 60% of the patients healed without needing further surgical intervention. 

In our study, no significant association was identified between the success of LIPUS therapy and the type of non-union, i.e., hypertrophic or atrophic non-union (p=0.449) (Table 1). As hypertrophic non-union is generally believed to be a consequence of inadequate stabilization of a fracture site [19], one would not expect LIPUS therapy to produce positive results. This type of non-union would traditionally be treated with revision surgery to achieve adequate reduction and fixation. In the systematic review by Leighton et al., the contrary was shown, with evidence that hypertrophic non-unions benefited more from LIPUS than atrophic non-unions [5].

Our study identified that the patients treated with initial surgical fixation had a higher success rate than patients treated conservatively, although this was not statistically significant (p=0.276). This could be attributed to many factors, of which the most likely factor would be the interfragmentary bone gap. This was proven to be a statistically significant factor influencing the success of LIPUS therapy in this study population (p=0.003). NICE also recognizes the importance of the interfragmentary bone gap as they advised that only patients with a gap of 10mm or less should be considered for LIPUS therapy [12]. Furthermore, the statistical significance of the interfragmentary bone gap has also been demonstrated by many other publications [17,20-23].

The timing of initiating LIPUS therapy should also be considered. It has been suggested that starting LIPUS therapy more than six months after the last surgery or initial injury could reduce union rates [24]. This is also the case in this study, with an average time to initiating LIPUS of 4.4 (SD±2.3) months in the successful treatment group. In contrast, the failed LIPUS treatment group only initiated therapy at 6.6 (SD±5.2) months on average.

When considering the sociodemographic background of patients being treated with LIPUS therapy, one would expect an apparent association between specific risk factors and treatment success. This has not been the case when reviewing the available literature. Some studies do suggest that the effectiveness of LIPUS is negatively impacted by age [25], smoking [11,26], and Diabetes [27], whereas others studies contradict this [5,21,24,25,28]. This study also demonstrated no significant association between the success of LIPUS therapy and the patient's sex, age, diabetes, and smoking history (Table 1).

Limitations of this study includes: a retrospective study without a non-LIPUS treatment or placebo arm; no standardization about decision-making and timing to initiate LIPUS therapy; multiple different fracture sites, patterns, and initial treatment methods included.

However, this study is more extensive than many recently published studies. The findings reflect the attitude of a large department with significant pressures on theatre access, where most consultants view LIPUS as a low-risk treatment tool and are open to its use.

## Conclusions

This study demonstrated a 61.6% progression to union rate of patients treated with LIPUS therapy for delayed union and non-union. Smaller interfragmentary bone gaps were identified as the only statistically significant factor positively influencing the success of LIPUS therapy. With patients initiated earlier on LIPUS therapy demonstrated a statistically non-significant improved success rate. No statistically significant association was identified between the success of LIPUS therapy and the patient's age, sex, diabetes, and smoking status.

Although the union rates demonstrated in this study could be considered low, it is still important to note that more than 60% of the patients healed without needing further surgical intervention. In the current climate post lockdown and ongoing spread of Covid-19, there is an increasing value and demand for non-surgical treatment options, with an ever-increasing elective surgery waiting list. LIPUS therapy represents an important complementary non-surgical and low-risk treatment pathway for delayed union and non-union. Furthermore, LIPUS therapy plays a vital role in treating complicated cases, with patients who might have significant co-morbidities and would otherwise not be suitable surgical candidates.

## References

[1] Langdahl B, Ferrari S, Dempster DW (2016). Bone modeling and remodeling: potential as therapeutic targets for the treatment of osteoporosis. Ther Adv Musculoskelet Dis.

[2] Zura R, Xiong Z, Einhorn T (2016). Epidemiology of fracture nonunion in 18 human bones. JAMA Surg.

[3] Zura R, Kaste SC, Heffernan MJ, Accousti WK, Gargiulo D, Wang Z, Steen RG (2018). Risk factors for nonunion of bone fracture in pediatric patients: an inception cohort study of 237,033 fractures. Medicine (Baltimore).

[4] Clark D, Nakamura M, Miclau T, Marcucio R (2017). Effects of aging on fracture healing. Curr Osteoporos Rep.

[5] Leighton R, Watson JT, Giannoudis P, Papakostidis C, Harrison A, Steen RG (2017). Healing of fracture nonunions treated with low-intensity pulsed ultrasound (LIPUS): a systematic review and meta-analysis. Injury.

[6] Harrison A, Alt V (2021). Low-intensity pulsed ultrasound (LIPUS) for stimulation of bone healing - a narrative review. Injury.

[7] Copuroglu C, Calori GM, Giannoudis PV (2013). Fracture non-union: who is at risk?. Injury.

[8] Gebauer D, Mayr E, Orthner E, Ryaby JP (2005). Low-intensity pulsed ultrasound: effects on nonunions. Ultrasound Med Biol.

[9] Ekegren CL, Edwards ER, de Steiger R, Gabbe BJ (2018). Incidence, costs and predictors of non-union, delayed union and Mal-Union following long bone fracture. Int J Environ Res Public Health.

[10] Duarte LR (1983). The stimulation of bone growth by ultrasound. Arch Orthop Trauma Surg (1978).

[11] Rutten S, Nolte PA, Korstjens CM, van Duin MA, Klein-Nulend J (2008). Low-intensity pulsed ultrasound increases bone volume, osteoid thickness and mineral apposition rate in the area of fracture healing in patients with a delayed union of the osteotomized fibula. Bone.

[12] Higgins A, Glover M, Yang Y, Bayliss S, Meads C, Lord J (2014). EXOGEN ultrasound bone healing system for long bone fractures with non-union or delayed healing: a NICE medical technology guidance. Appl Health Econ Health Policy.

[13] Bashardoust Tajali S, Houghton P, MacDermid JC, Grewal R (2012). Effects of low-intensity pulsed ultrasound therapy on fracture healing: a systematic review and meta-analysis. Am J Phys Med Rehabil.

[14] Leighton R, Phillips M, Bhandari M, Zura R (2021). Low intensity pulsed ultrasound (LIPUS) use for the management of instrumented, infected, and fragility non-unions: a systematic review and meta-analysis of healing proportions. BMC Musculoskelet Disord.

[15] Hughes LD, Khudr J, Gee E, Pillai A (2022). Pitfalls preventing bone union with EXOGEN low-intensity pulsed ultrasound. SICOT J.

[16] Adukia V, Al-Hubeshy Z, Mangwani J (2021). Can low intensity pulsed ultrasound (LIPUS) be used as an alternative to revision surgery for patients with non-unions following fracture fixation?. J Clin Orthop Trauma.

[17] Biglari B, Yildirim TM, Swing T, Bruckner T, Danner W, Moghaddam A (2016). Failed treatment of long bone nonunions with low intensity pulsed ultrasound. Arch Orthop Trauma Surg.

[18] Schofer MD, Block JE, Aigner J, Schmelz A (2010). Improved healing response in delayed unions of the tibia with low-intensity pulsed ultrasound: results of a randomized sham-controlled trial. BMC Musculoskelet Disord.

[19] Niikura T, Lee SY, Sakai Y, Nishida K, Kuroda R, Kurosaka M (2014). Causative factors of fracture nonunion: the proportions of mechanical, biological, patient-dependent, and patient-independent factors. J Orthop Sci.

[20] Roussignol X, Currey C, Duparc F, Dujardin F (2012). Indications and results for the Exogen™ ultrasound system in the management of non-union: a 59-case pilot study. Orthop Traumatol Surg Res.

[21] Watanabe Y, Arai Y, Takenaka N, Kobayashi M, Matsushita T (2013). Three key factors affecting treatment results of low-intensity pulsed ultrasound for delayed unions and nonunions: instability, gap size, and atrophic nonunion. J Orthop Sci.

[22] Claes L, Augat P, Suger G, Wilke HJ (1997). Influence of size and stability of the osteotomy gap on the success of fracture healing. J Orthop Res.

[23] Drosos GI, Bishay M, Karnezis IA, Alegakis AK (2006). Factors affecting fracture healing after intramedullary nailing of the tibial diaphysis for closed and grade I open fractures. J Bone Joint Surg Br.

[24] Jingushi S, Mizuno K, Matsushita T, Itoman M (2007). Low-intensity pulsed ultrasound treatment for postoperative delayed union or nonunion of long bone fractures. J Orthop Sci.

[25] Zura R, Mehta S, Della Rocca GJ, Jones J, Steen RG (2015). A cohort study of 4,190 patients treated with low-intensity pulsed ultrasound (LIPUS): findings in the elderly versus all patients. BMC Musculoskelet Disord.

[26] Teoh KH, Whitham R, Wong JF, Hariharan K (2018). The use of low-intensity pulsed ultrasound in treating delayed union of fifth metatarsal fractures. Foot (Edinb).

[27] Berber R, Aziz S, Simkins J, Lin SS, Mangwani J (2020). Low Intensity Pulsed Ultrasound Therapy (LIPUS): a review of evidence and potential applications in diabetics. J Clin Orthop Trauma.

[28] Elvey MH, Miller R, Khor KS, Protopapa E, Horwitz MD, Hunter AR (2020). The use of low-intensity pulsed ultrasound in hand and wrist nonunions. J Plast Surg Hand Surg.

